# Supplementary material for: Oxide-hybridized carbon as a catalyst support for efficient anion exchange membrane water electrolysis
Source: Nat Commun. 2025 Dec 12;16:11090. doi: 10.1038/s41467-025-65980-w (PMC12700888; doi:10.1038/s41467-025-65980-w)
Supplement: Supplementary file 1 — Supplementary Information [file 41467_2025_65980_MOESM1_ESM.pdf]

Supplementary Information

Oxide-Hybridized Carbon as a Catalyst Support for Efficient Anion Exchange Membrane Water Electrolysis

Jong Seok Park[1,2†], Hyung-Kyu Lim[3†], Chuan Hu[4,5†], Eungjun Lee[1], Hyo Sang Jeon[6], Jong Kyung Ryu[7], Sion Oh[1,8], Tae Kyung Lee[1,2], Subin Park[1], Hyeon Keun Cho[5], Seung-Ho Yu[2], Docheon Ahn[9], Young Moo Lee[5], Myeong-Geun Kim[1,10*], and Sung Jong Yoo[1,10*]*

[1]Center for Hydrogen and Fuel Cells, Korea Institute of Science and Technology (KIST), 5 Hwarang–ro 14–gil, Seongbuk–gu, Seoul 02792, Republic of Korea
[2]Department of Chemical and Biological Engineering, Korea University, 145, Anam-ro, Seongbuk-gu, Seoul 02841, Republic of Korea
[3]Division of Chemical and Bioengineering, Kangwon National University, 1, Kangwondaehak-gil, Chuncheon, Gangwon-do 24341 Republic of Korea
[4]School of Energy and Environment, Southeast University, No. 2, Southeast University Road, Jiangning District, Nanjing, Jiangsu Province, China
[5]Department of Energy Engineering, Hanyang University, 222, Wangsimni-ro, Seongdong-gu, Seoul 04763, Republic of Korea
[6]Sustainable Energy Research Division, Korea Institute of Science and Technology (KIST), 5 Hwarang–ro 14–gil, Seongbuk–gu, Seoul 02792, Republic of Korea
[7]Department of Materials Science and Engineering, Pohang University of Science and Technology (POSTECH) Pohang, Gyeongbuk 37673, Republic of Korea
[8]Department of Chemical Engineering, Kyung Hee University, 1732 Deogyeong-daero, Giheung-gu, Yongin-si, Gyeonggi-do 17104, Republic of Korea
[9]Beamline Division, Pohang Accelerator Laboratory, 80, Jigok-ro 127 beon-gil, Nam-gu, Pohang 37673, Republic of Korea
[10]Division of Energy & Environment Technology, KIST School, University of Science and Technology (UST), Seoul 02792, Republic of Korea

**Corresponding Author:*

Sung Jong Yoo; E–mail: ysj@kist.re.kr

Myeong–Geun Kim; E–mail: mgkim@kist.re.kr

Young Moo Lee; E-mail: ymlee@hanyang.ac.kr

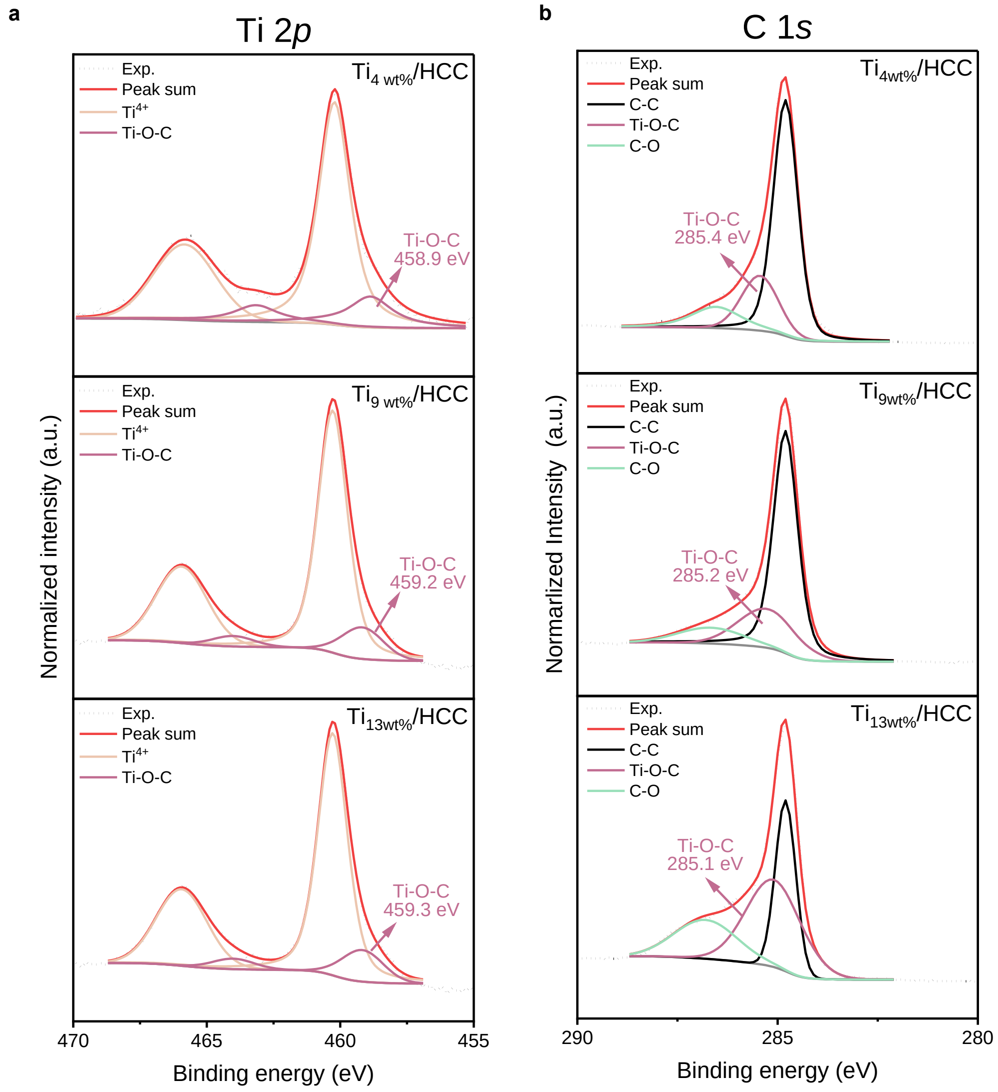

Fig. S1 | XPS (a) Ti 2*p* and (b) C 1*s* spectra for $Ti_{xwt\%}$/HCC

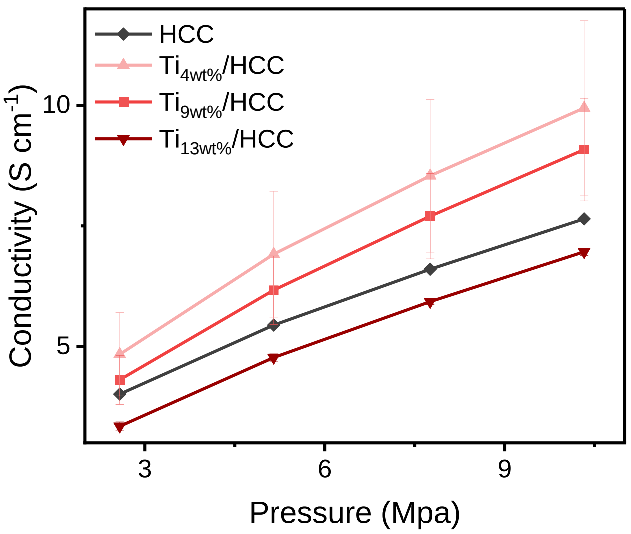

Fig. S2 | Electrical conductivity of $Ti_{xwt\%}$/HCC (error bars represent standard deviation from three independent measurement)

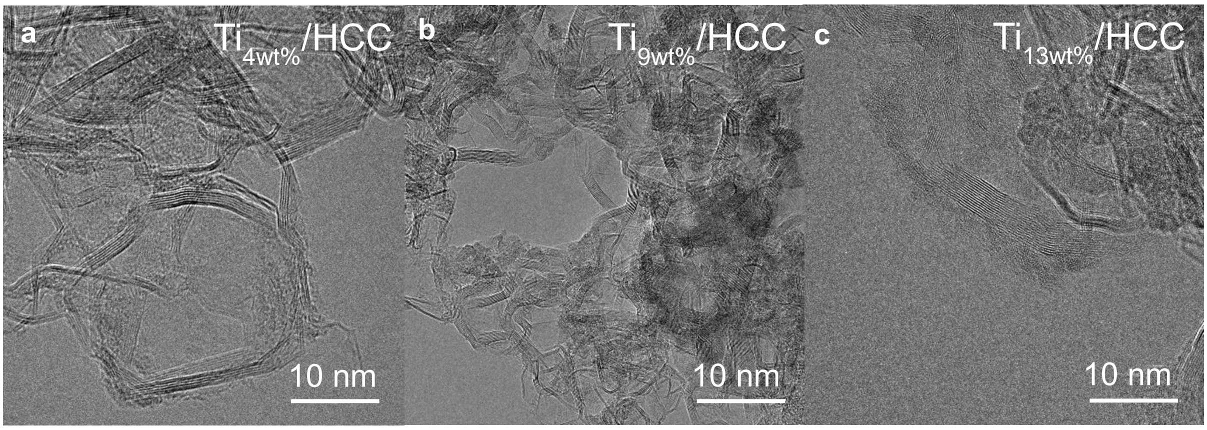

Fig. S3 | TEM images of (a) $Ti_{4wt\%}$/HCC, (b) $Ti_{9wt\%}$/HCC, and (c) $Ti_{13wt\%}$/HCC carbon

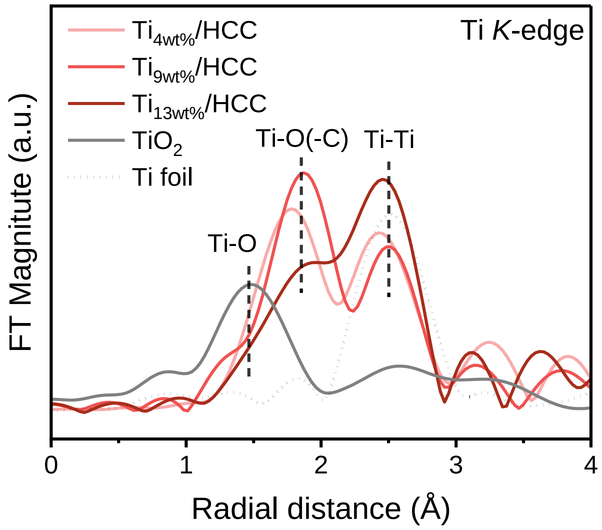

Fig. S4 | EXAFS spectra of the Ti *K*-edge of Ti/HCC

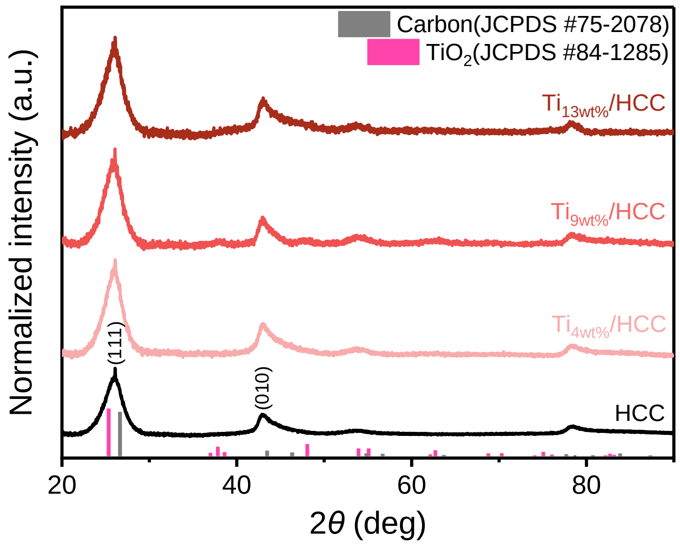

Fig. S5 | XRD pattern of $Ti_{xwt\%}$/HCC. The black and pink bar represent reference data for TiO_2 and carbon, respectively

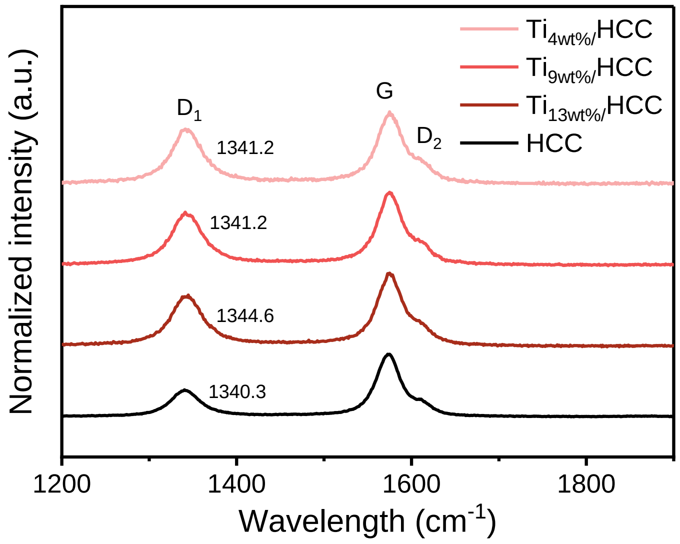

Fig. S6 | Raman spectra for $Ti_{xwt\%}$/HCC. The position of G peak was fixed at 1575 cm^{-1}

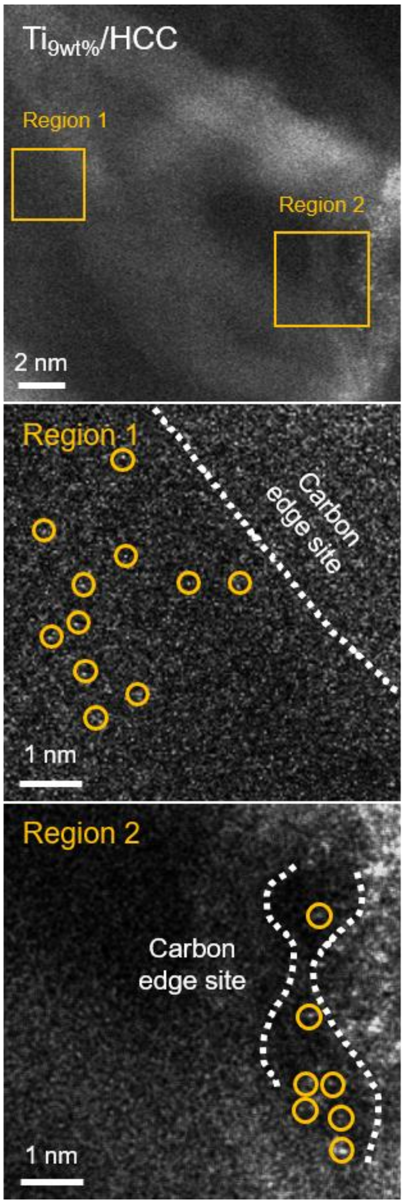

Fig. S7 | HR-STEM images of the $Ti_{9wt\%}$/HCC. Ti single atoms are marked with yellow circles

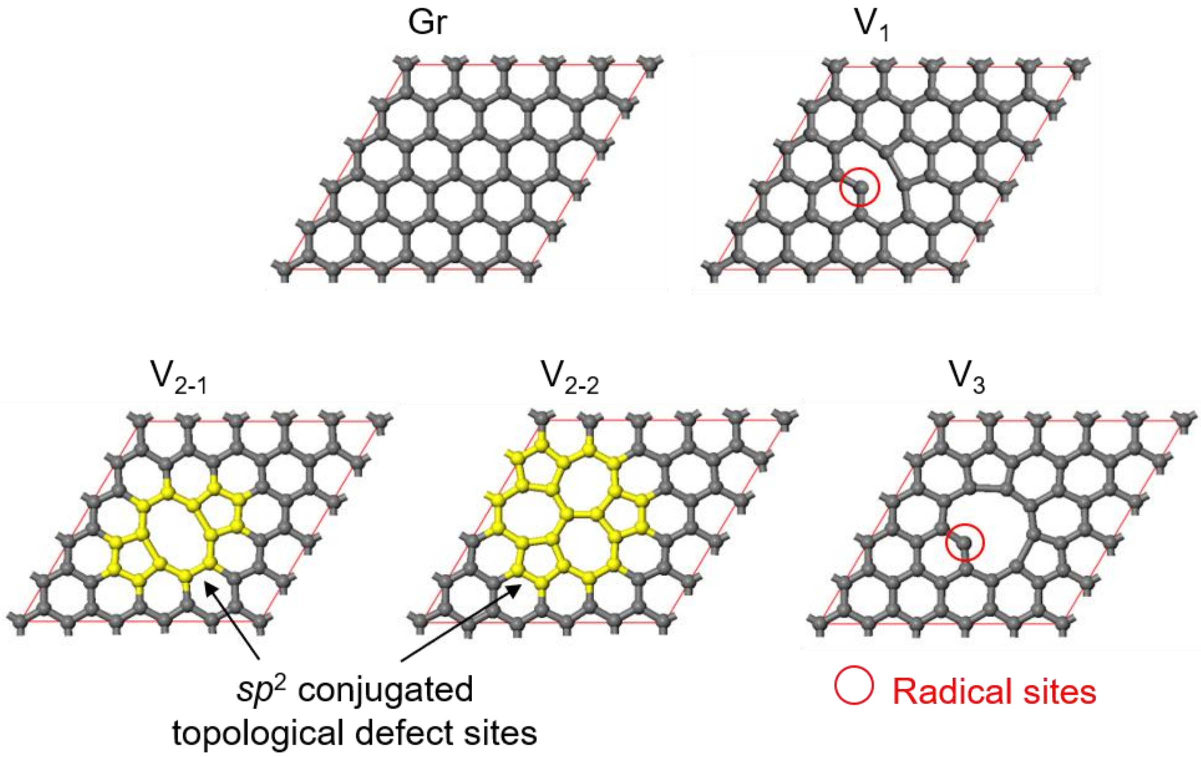

Fig. S8 | Optimized atomic structures showing different types of defect configurations in HCC: perfect graphene (Gr), radical defect sites (V_1, V_3) and *sp*^{2}-conjugated topological defect sites ($V_{2\text{-}1}$, $V_{2\text{-}2}$)

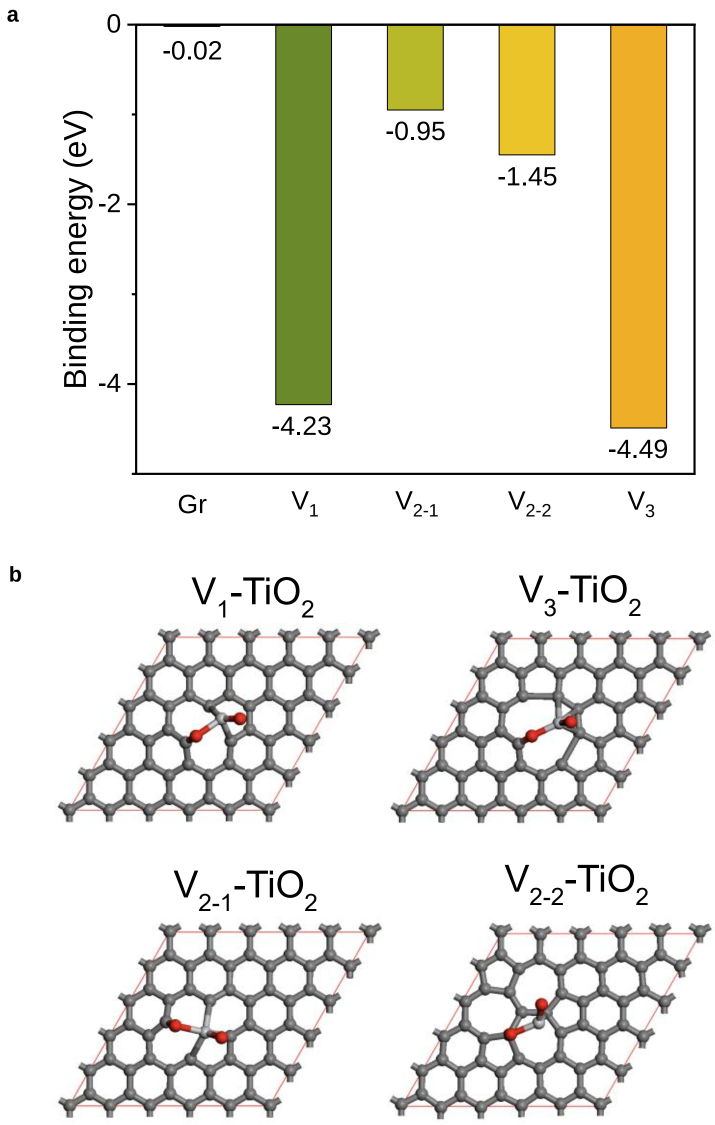

Fig. S9 | (a) Binding energy calculations for TiO_2 moiety at (b) different defect configurations of HCC, demonstrating preferential binding at radical defect sites

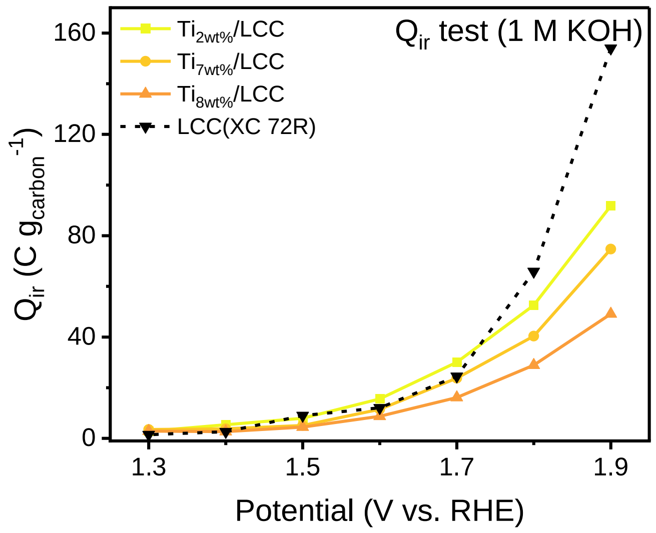

Fig. S10 | Q_{ir} test results for $Ti_{xwt\%}$/LCC (low crystalline carbon)

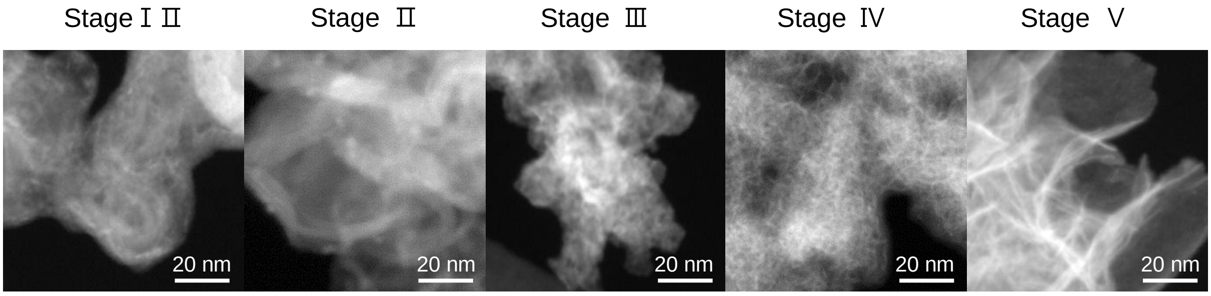

Fig. S11 | (a) *Ex*-situ STEM images of LDH-$Ti_{9wt\%}$/HCC obtained at each stage

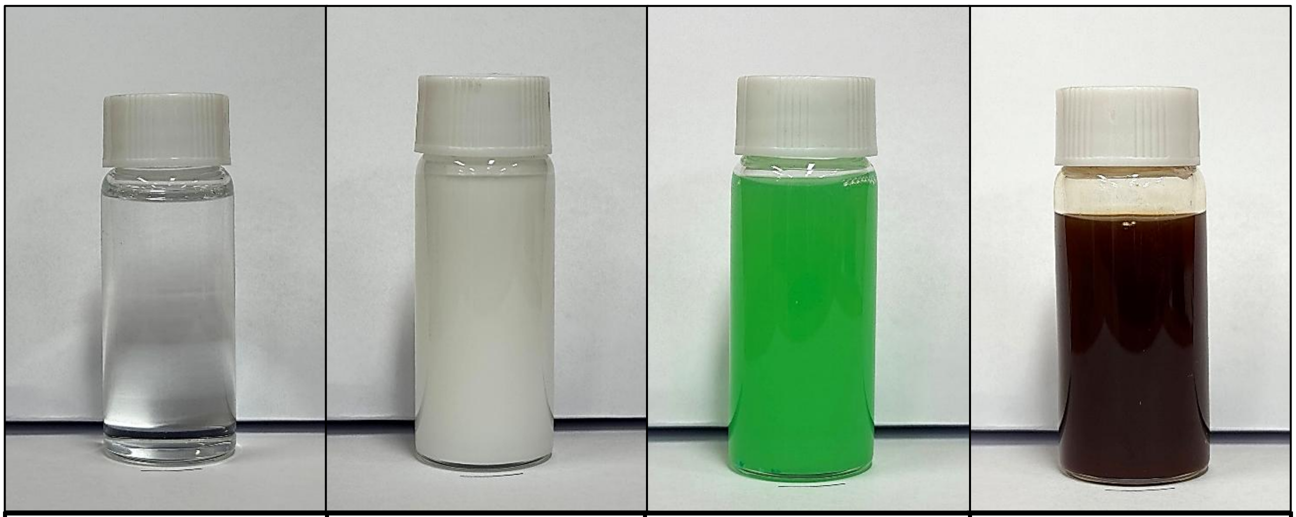

EG	EG + TTIP	EG + TTIP<br>+ Ni sulfate	EG + TTIP<br>+ Fe sulfate

Fig. S12 | Changes in the transparency of TTIP/EG solution depending on the type of injected metal precursor

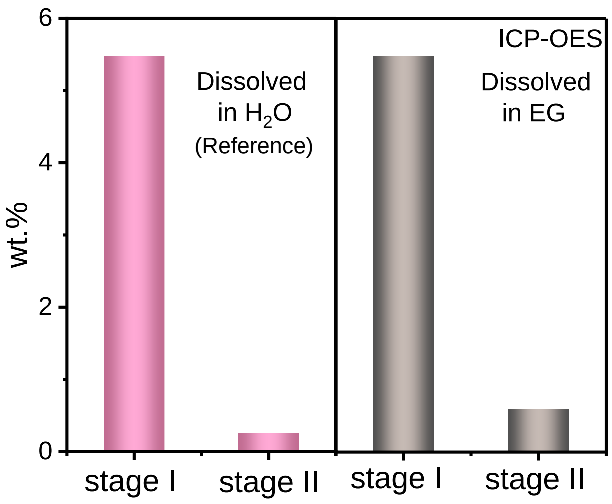

Fig. S13 | Comparison of Ti dissolution measured by ICP-OES based on the solution used for the injected metal precursor: H_2O (pink, reference) and EG (black)

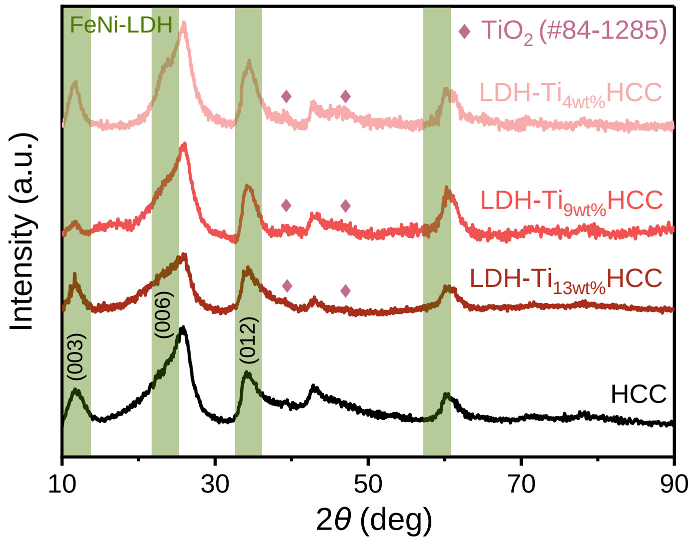

Fig. S14 | XRD patterns of LDH-Ti$_{xwt\%}$/HCC

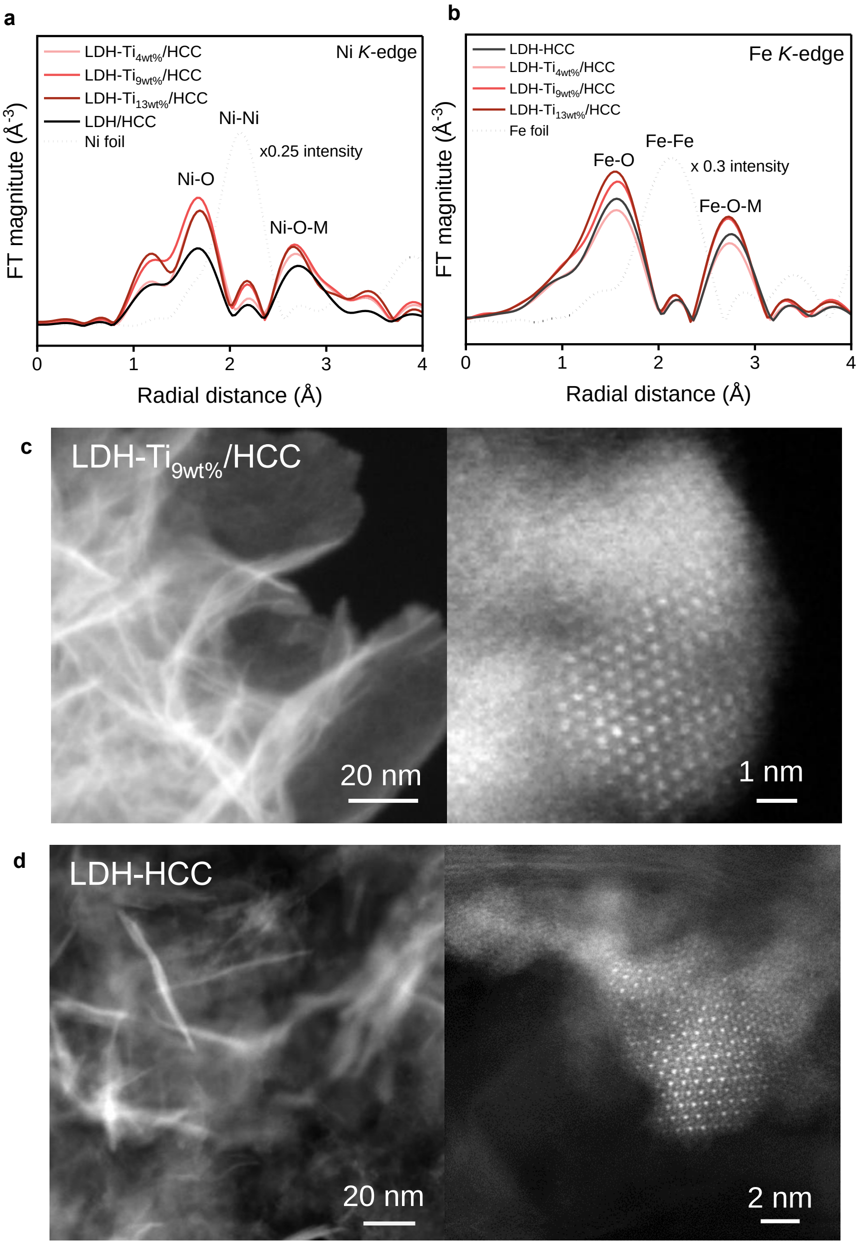

Fig. S15 | EXAFS spectra of (a) Ni *K*-edge and (b) Fe *K*-edge. HR-STEM images of (c) LDH-$Ti_{9wt\%}$/HCC and (d) LDH-HCC, respectively

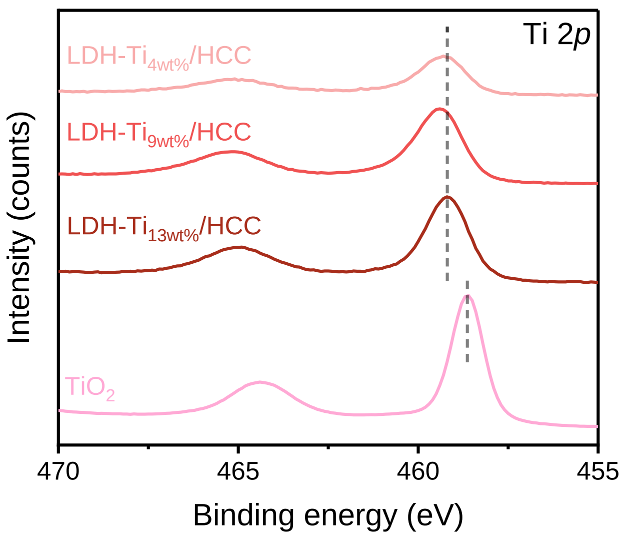

Fig. S16 | XPS Ti 2*p* spectra for LDH-$Ti_{xwt\%}$/HCC and commercial TiO_2

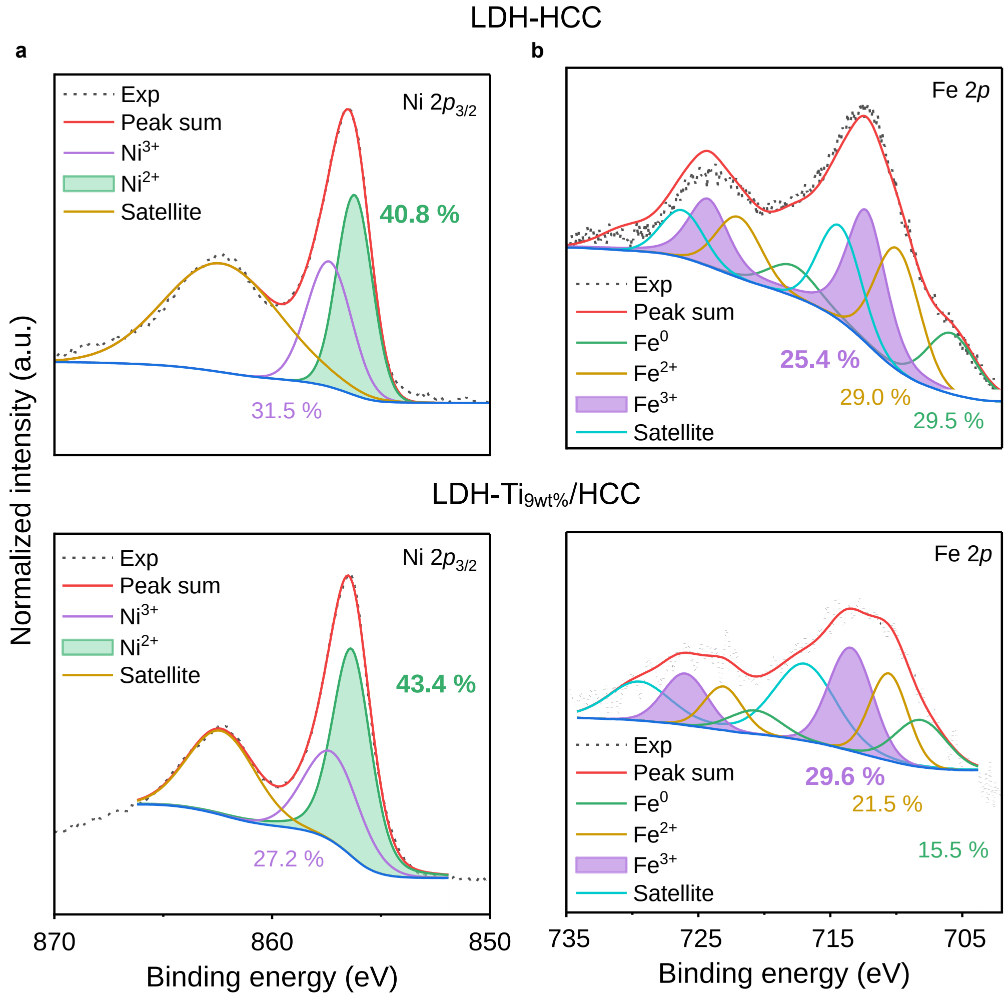

Fig. S17 | XPS spectra of (a) Ni 2*p* and (b) Fe 2*p* for LDH-$Ti_{9wt\%}$/HCC and LDH-HCC

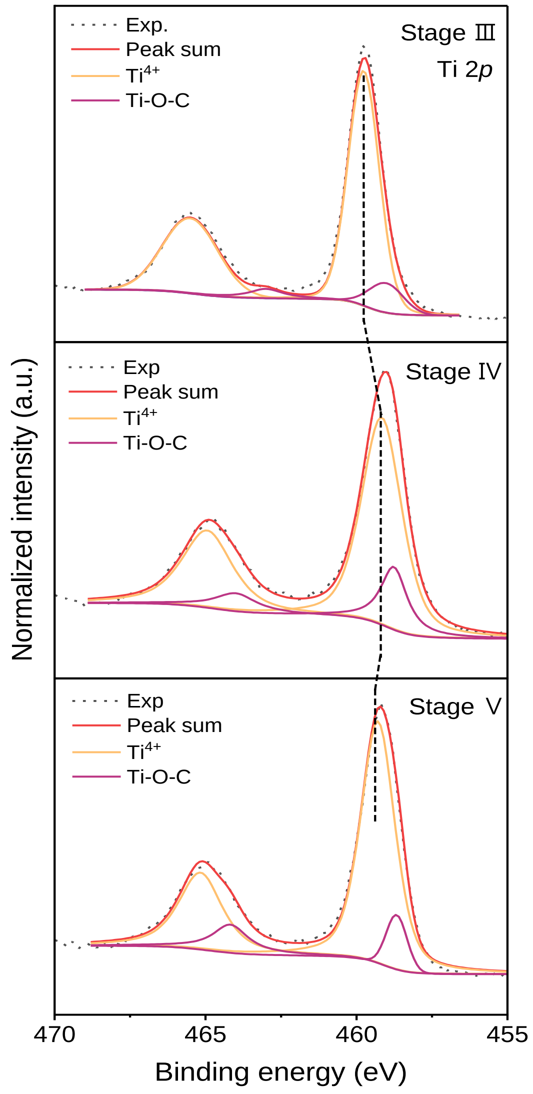

Fig. S18 | XPS spectra Ti 2*p* obtained at each stage

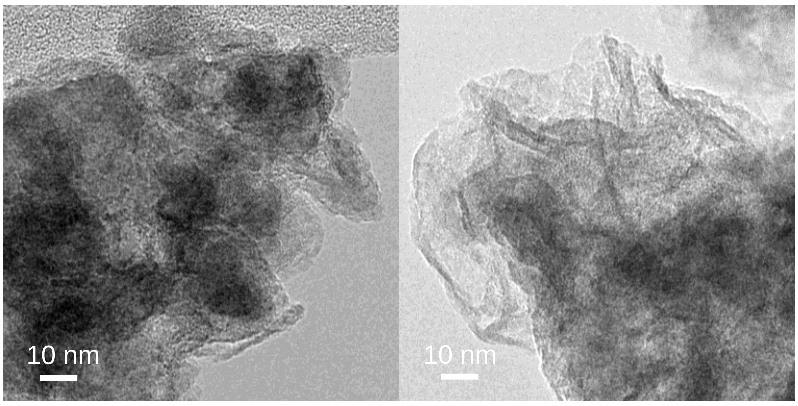

Fig. S19 | TEM image of unsupported FeNi LDH

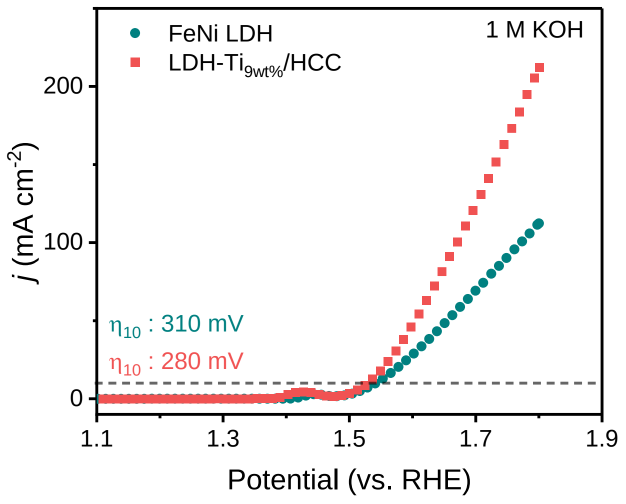

Fig. S20 | LSV curve for unsupported FeNi LDH and LDH-$Ti_{9wt\%}$/HCC

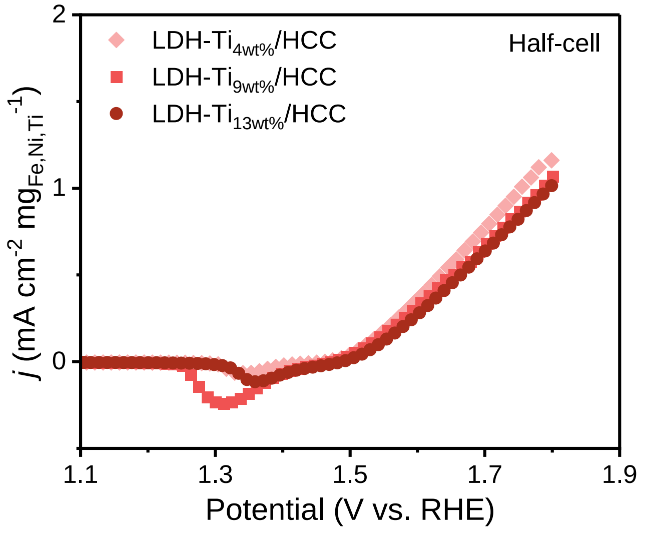

Fig. S21 | Potential dependent current density normalized with total metal loading amount

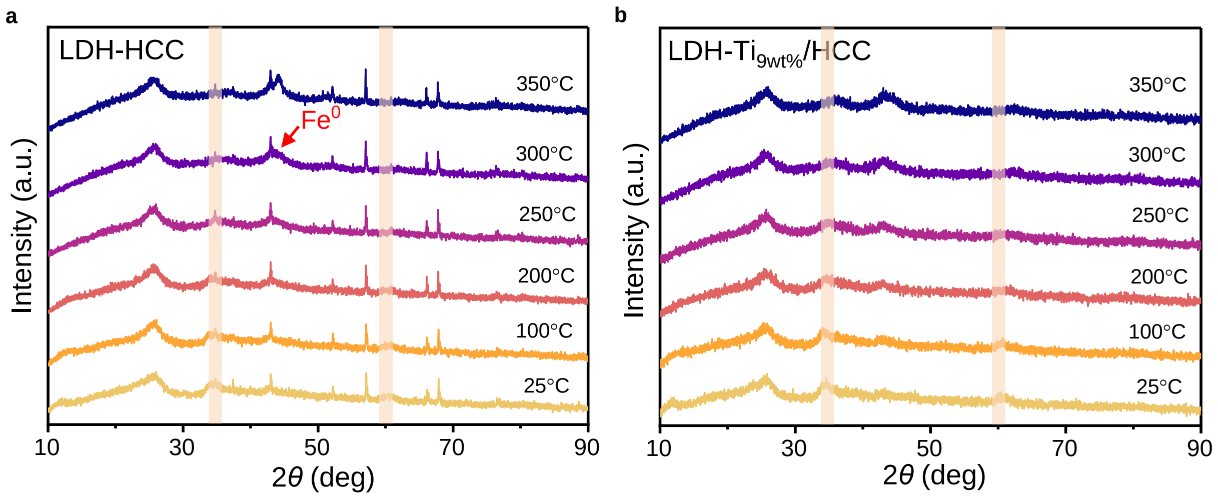

Fig. S22 | *In*-situ XRD patterns for (a) LDH/HCC and (b) LDH-$Ti_{9wt\%}$/HCC measured under heating conditions ranging from 25 to 350°C

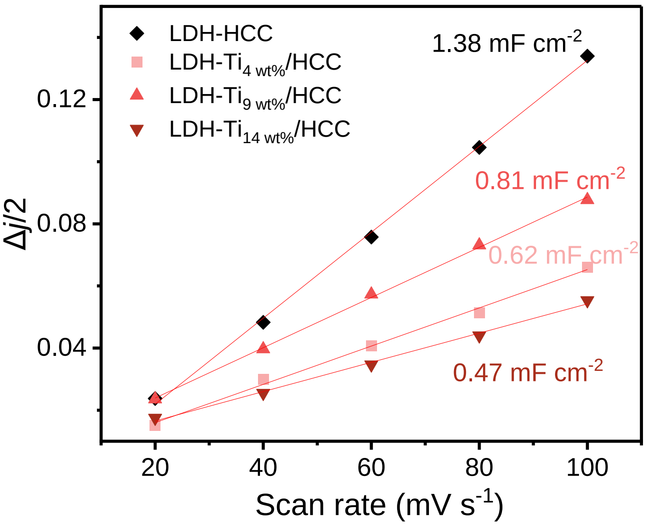

Fig. S23 | Electrochemical active surface area (ECSA) of LDH-$Ti_{xwt\%}$/HCC

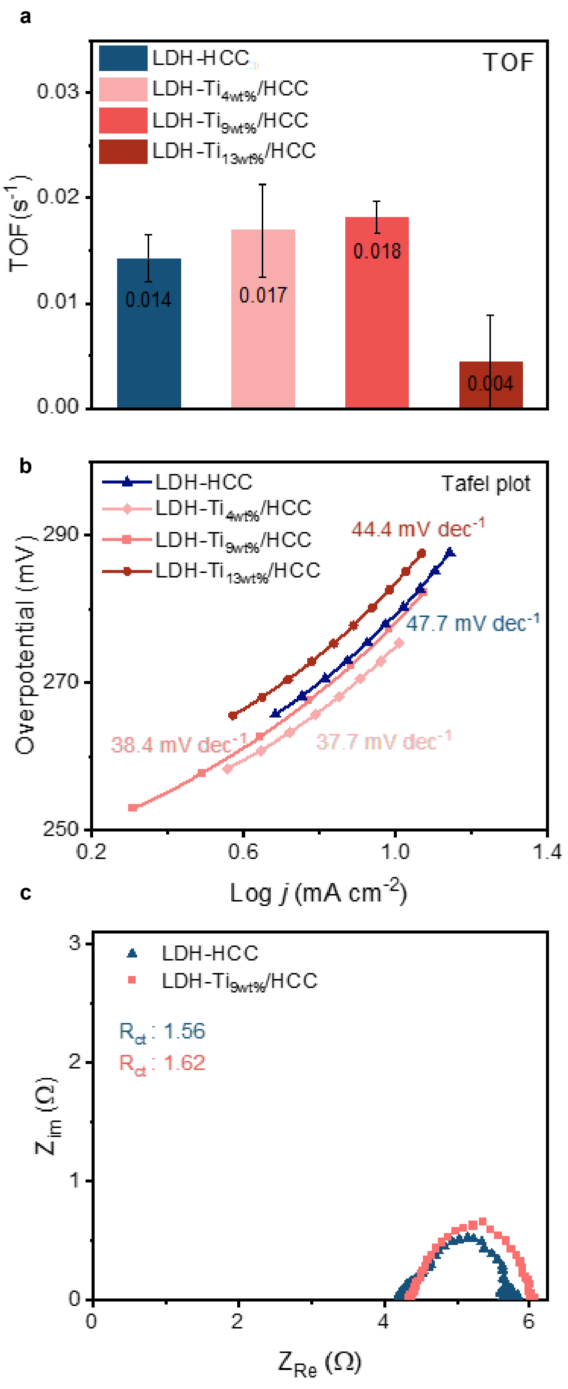

Fig. S24 | (a) TOF values, (b) Tafel plots, and (c) Nyquist plots

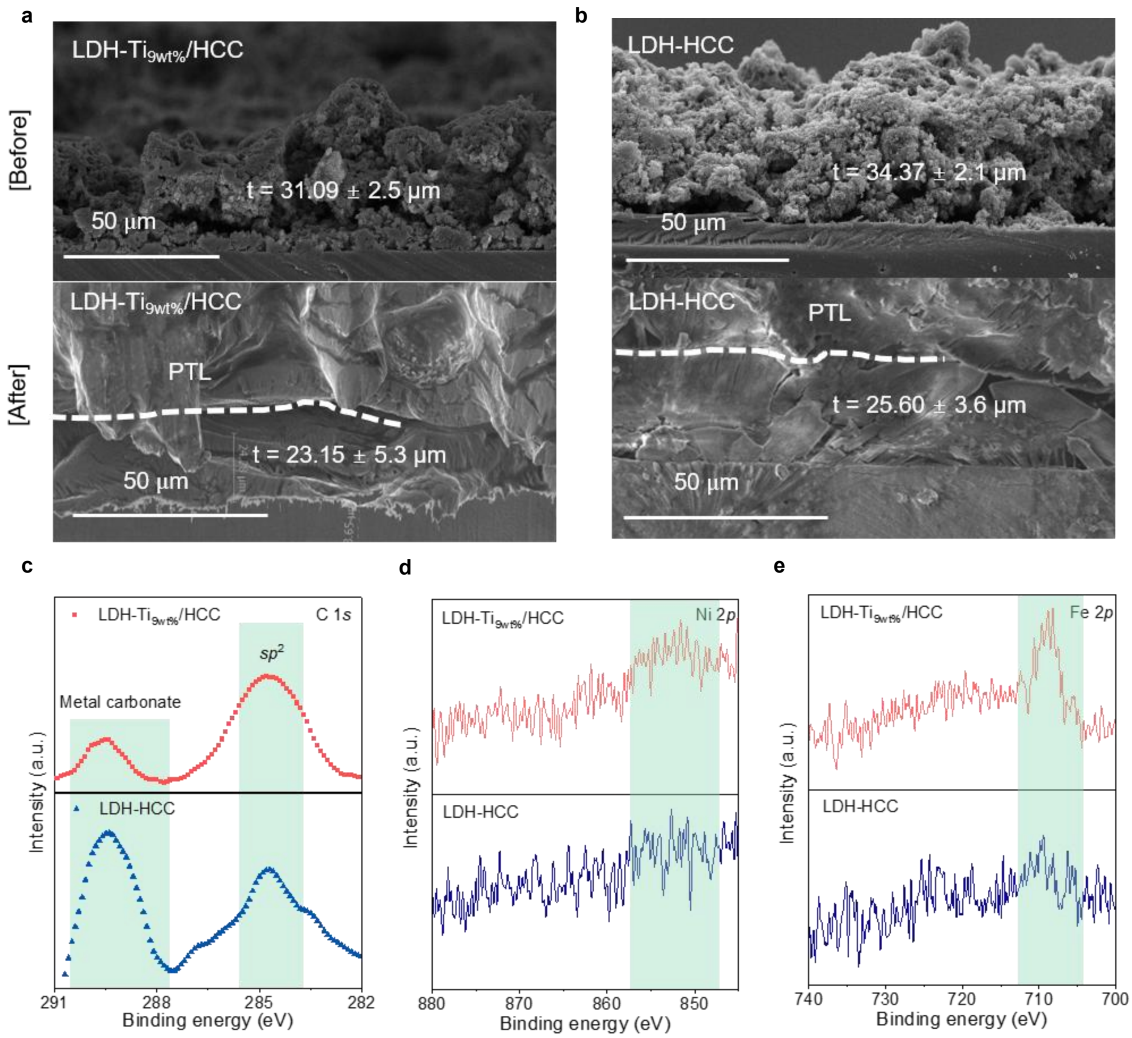

Fig. S25 | Cross-sectional SEM images of the MEA before and after a 200 h of operation: (a) LDH-Ti_{9wt}%/HCC and (b) LDH-HCC. (c) C 1*s*, (d) Ni 2*p*, and (e) Fe 2*p* XPS after the durability test

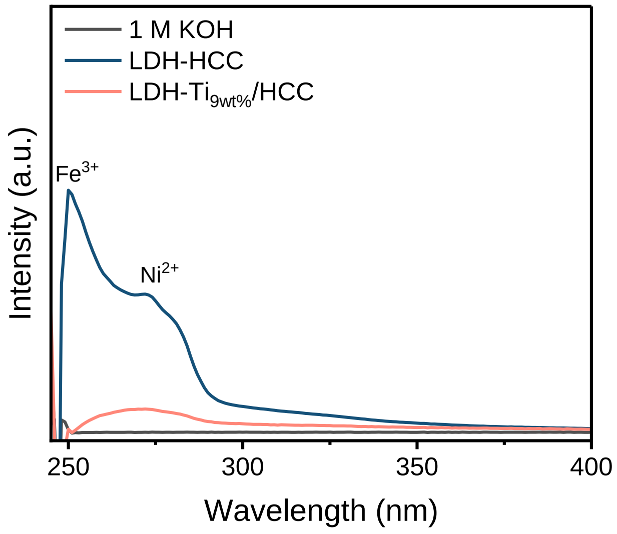

Fig. S26 | UV-vis spectra of the electrolyte collected after 1K cycles CV in the voltage region from 1.1 to 1.8 V_{RHE}

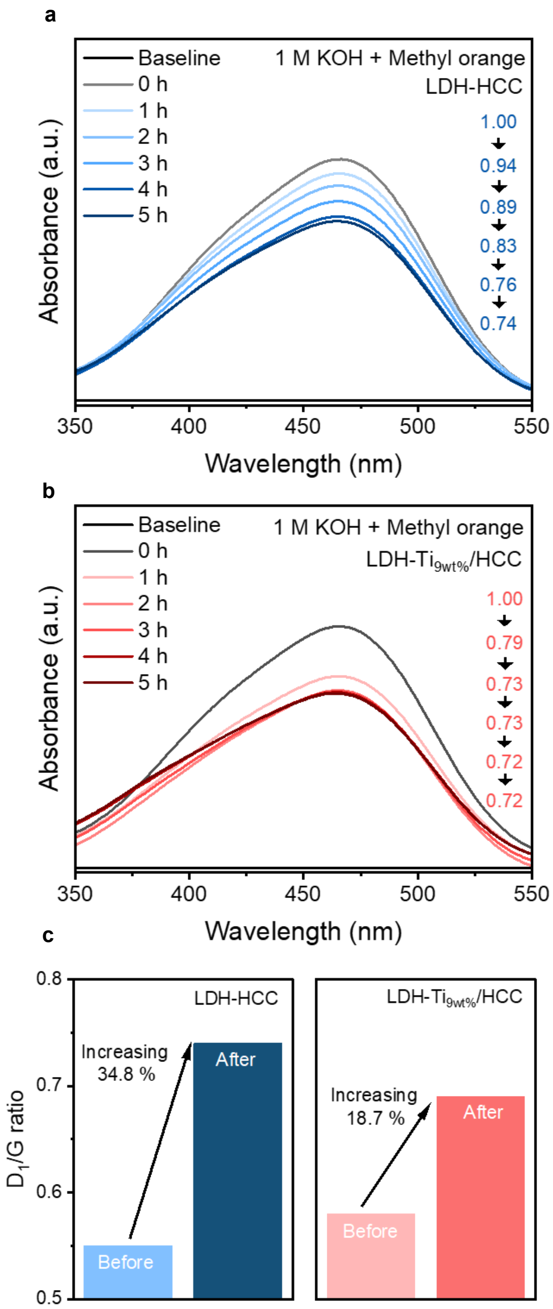

Fig. S27 | UV-vis spectra of the electrolyte containing methyl orange solution: (a) LDH-HCC and (b) LDH-$Ti_{9wt\%}$/HCC. (c) The D_1/G ratio before and after 5 h of reaction

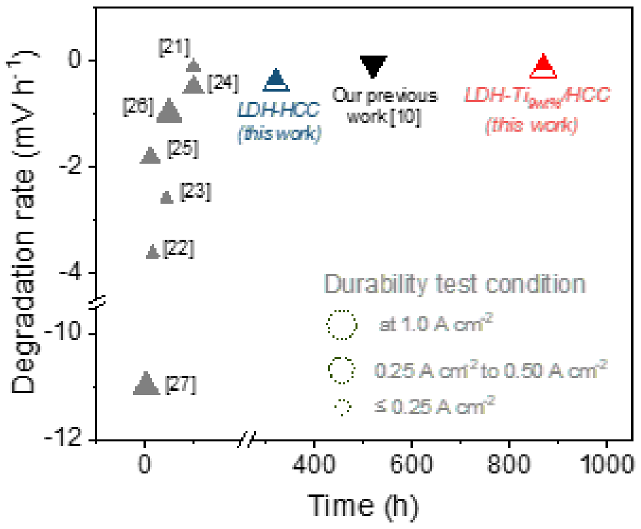

Fig. S28 | Durability comparison of this work with previously reported AEMWE catalysts.

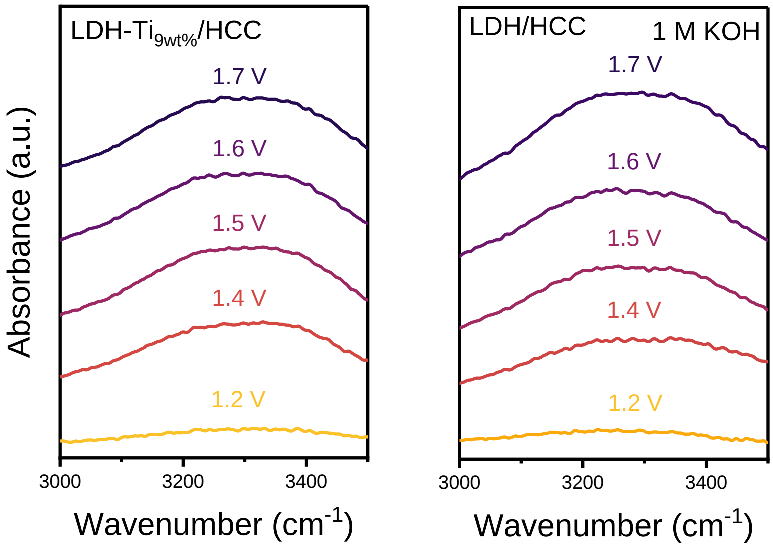

Fig. S29 | *In*-situ FT-IR measured from 1.2 to 1.7 V_{RHE} in 1 M KOH

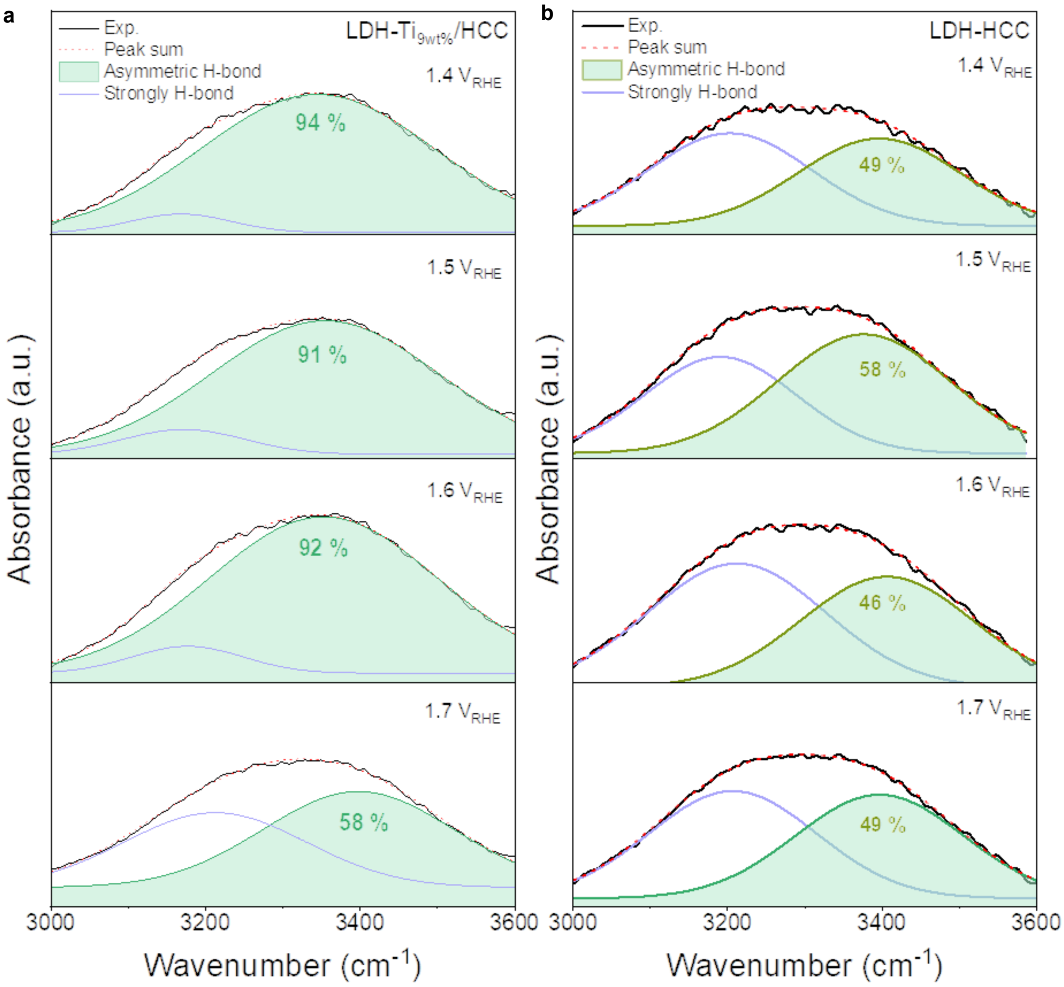

Fig. S30 | Deconvoluted *in*-situ FT-IR spectra for (a) LDH-$Ti_{9wt\%}$/HCC and (b) LDH- HCC.

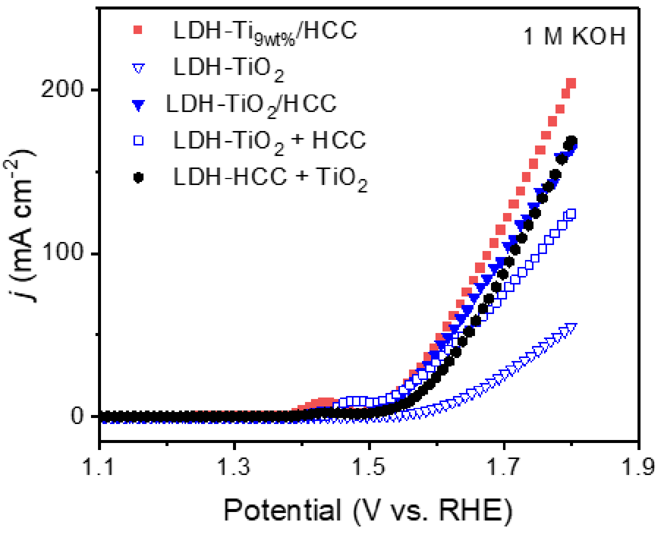

Fig. S31 | LSV curves of LDHs supported on different supporting materials: solid symbols represent LDH on carbon-based supports, while hollow symbols represent LDH on TiO_2.

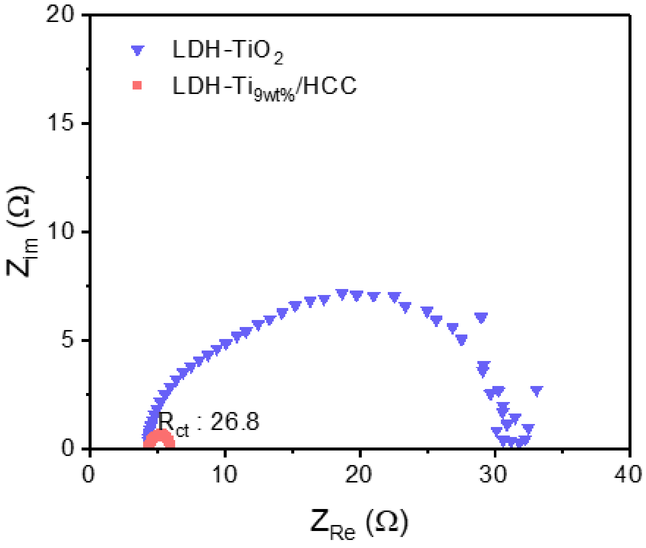

Fig. 32 | Nyquist plots of LDH-TiO_2 and LDH-$Ti_{9wt\%}$/HCC.

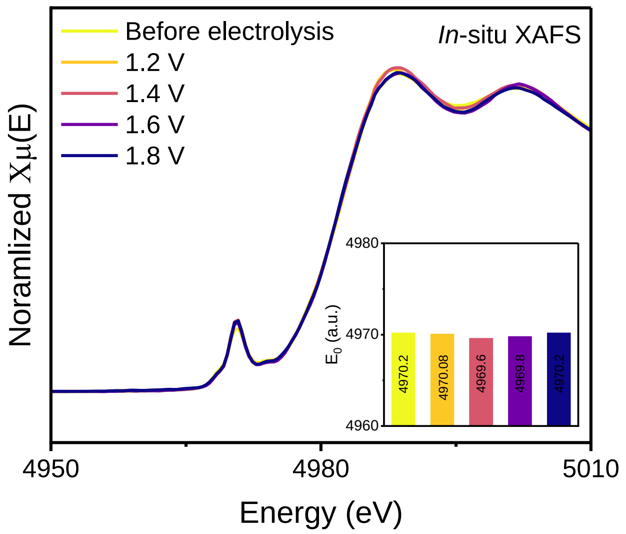

Fig. S33 | Quasi *in*-situ Ti K-edge XAFS spectra of LDH-$Ti_{9wt\%}$/HCC measured at different potentials: 1.2, 1.4, 1.6, and 1.8 V_{RHE}

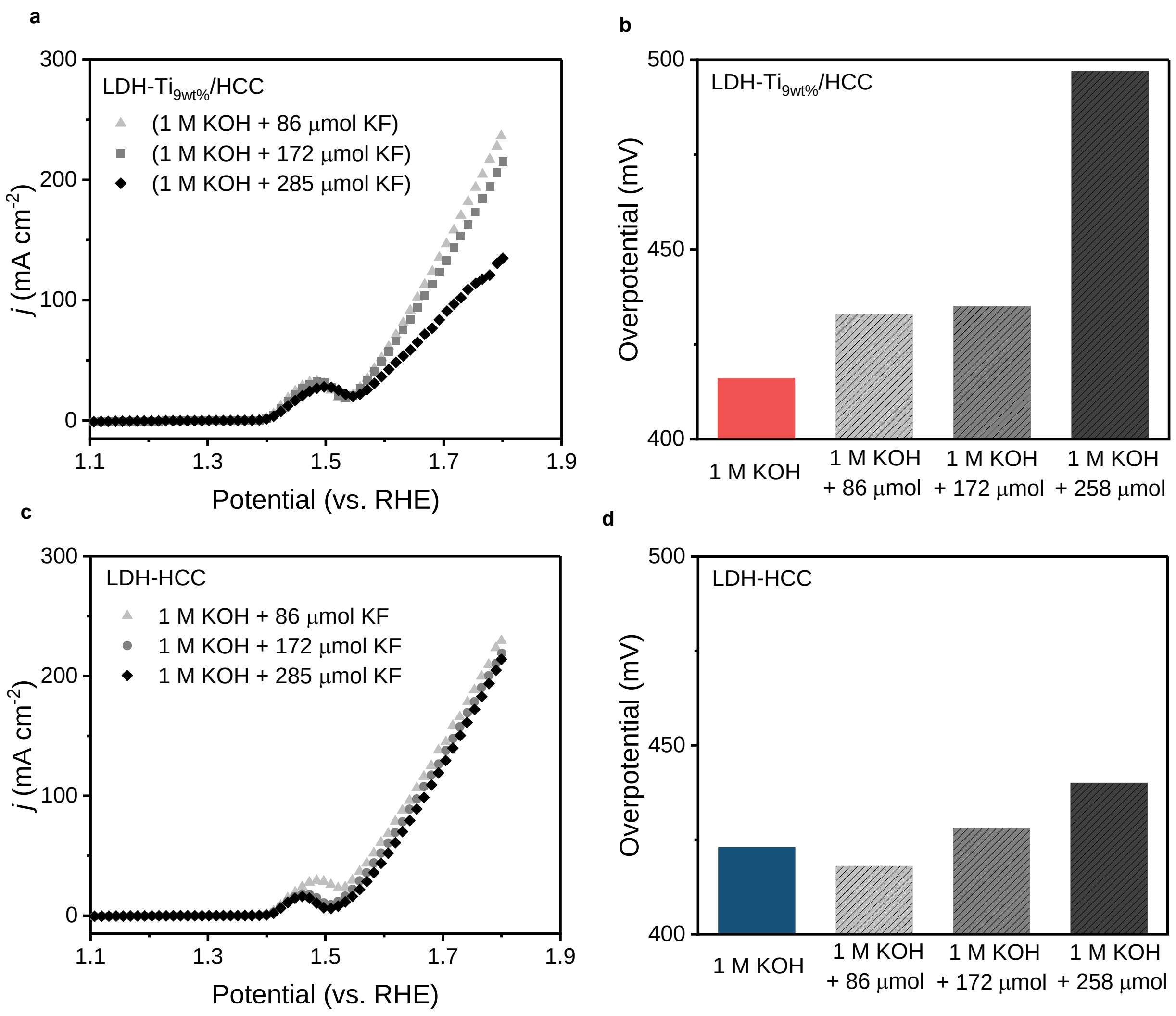

Fig. S34 | (a) LSV curve with different concentration of KF in 1M KOH and the corresponding increase in overpotential at 100 mA cm^{-2}: (a, b) LDH-$Ti_{9wt\%}$/HCC, (c, d) LDH-HCC. LSV curves were measured after 100 CV cycles in the range of 1.1 to 1.8 V_{RHE}, allowing sufficient time for F^- ions to poison the catalyst surface

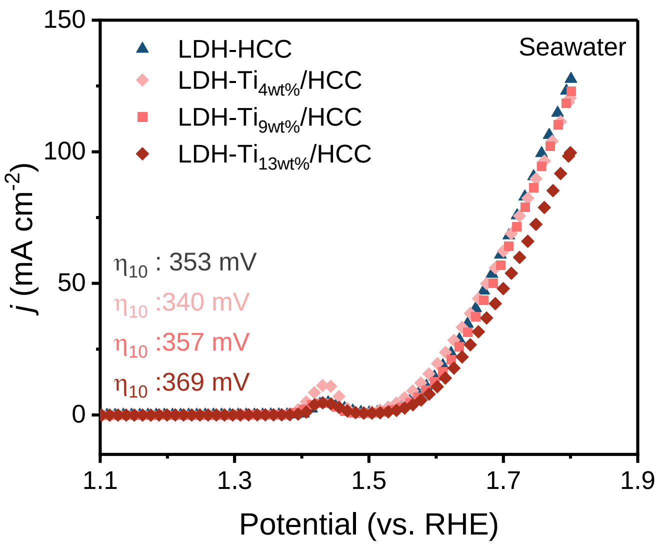

Fig. S35 | LSV curve under seawater conditions

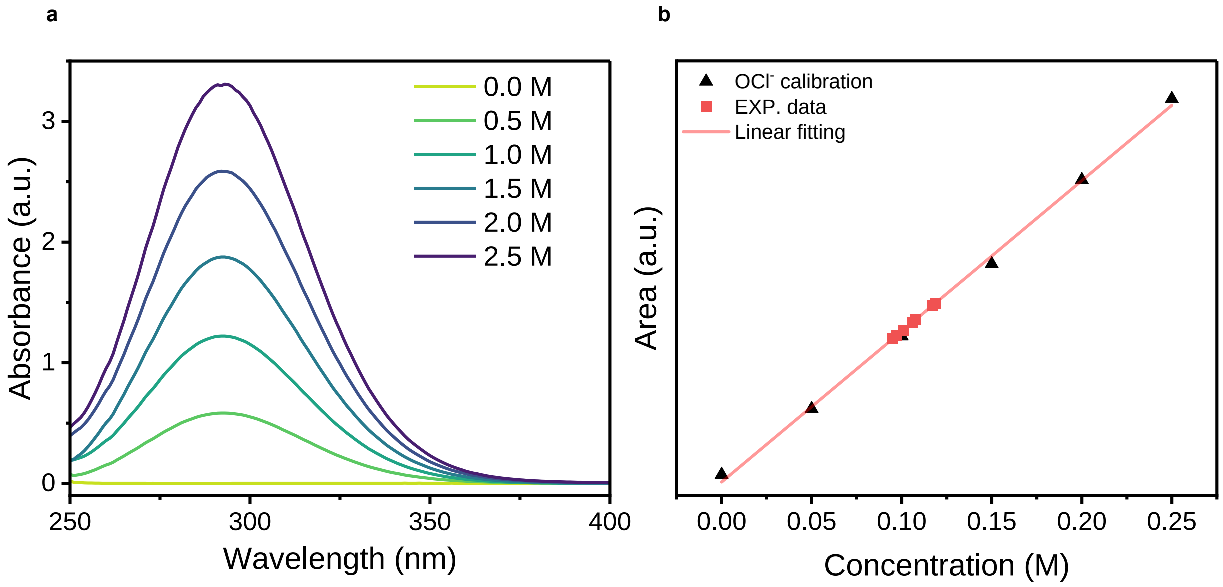

Fig. S36 | (a) UV-Vis spectra of 1 M KOH with varying NaCl concentrations (0.0 to 2.5 M), and (b) the corresponding calibration curve

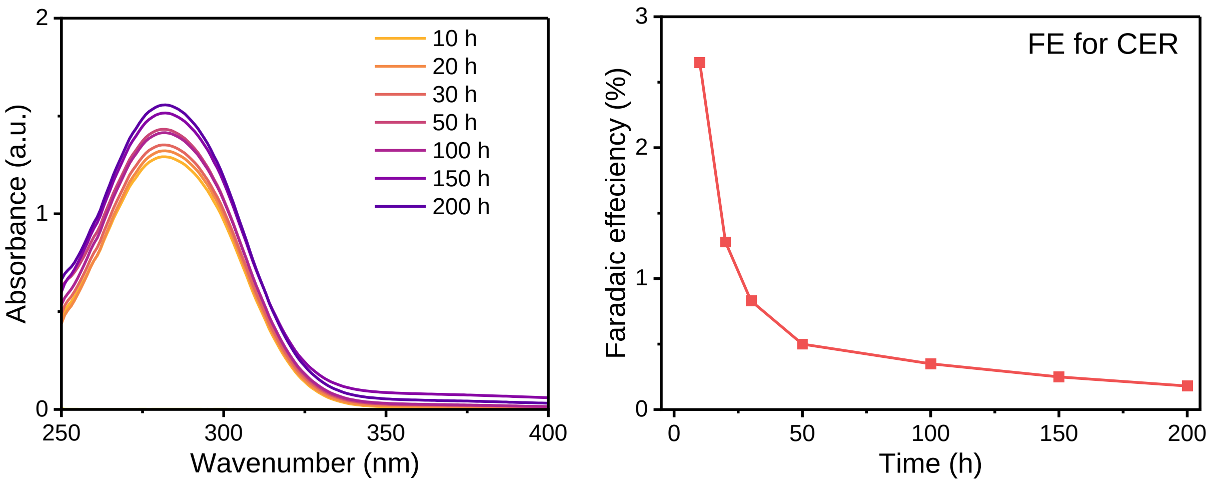

Fig. S37 | (a) UV-vis spectra of aliquots extracted from the electrolyte during the long-term durability test and (b) corresponding CER selectivity

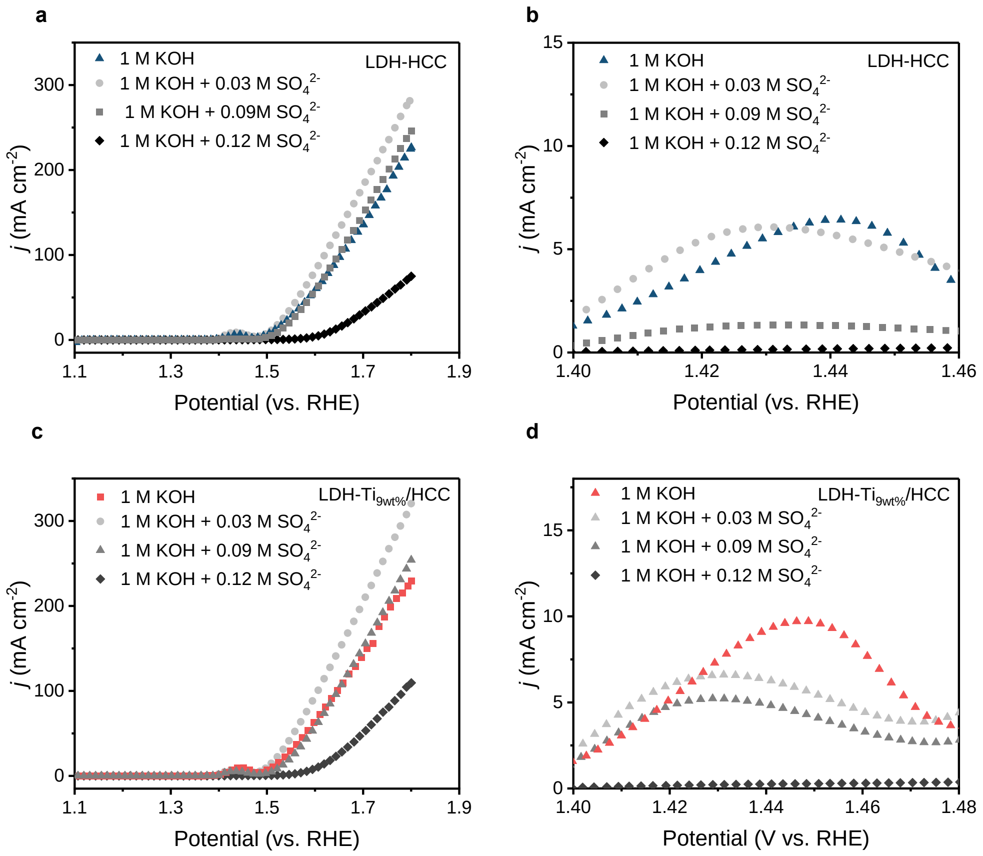

Fig. S38 | LSV curves obtained at different concentration of SO_4^{2-} in 1M KOH, showing changes in the peak area corresponding to Ni oxidation: (a, b) LDH-HCC and (c, d) LDH-$Ti_{9wt\%}$/HCC. LSV curves were measured after 15 CV cycles in the range of 1.1 to 1.8 V_{RHE}, allowing sufficient time for SO_4^{2-} ions to poison the catalyst surface

Table S1. Summary table of AEMWE cell configuration for durability comparison

Catalyst loading [mg cm^{-2}]		Electrolyte	Membrane	PTL		Applied current [A cm^{-2}]	Temperature	Flow rate [mL min^{-1}]	Active area [cm^2]	Ref
Anode	Cathode			Anode	Cathode					
0.25	0.5	1 M KOH	PDTP	SUS	Carbon paper	1.0	80 ℃	36	5.0	This work
0.25	0.5	1 M KOH	PDTP	SUS	Carbon paper	1.0	80 ℃	36	5.0	This work
0.25	0.5	1 M KOH	PDTP	SUS	Carbon paper	1.0	80 ℃	36	5.0	[10]
1	0.4	1 M KOH	FAA-3-50	SUS	Carbon paper	0.01	70 ℃	1	5.0	[21]
10	Nickel foam	10 wt.% KOH	-	Nickel foam		0.225	50 ℃	350	4.0	[22]
0.4	0.38	1 M KOH	FAA-3-50	Carbon paper		0.25	40-70 ℃	2	6.25	[23]
10	1	1 M KOH	X37-50	Nickel foam	Carbon paper	0.4	50 ℃	50	4.9	[24]
23	1	1 M KOH	X37-50	Nickel foam		0.5	45 ℃	24	4.9	[25]
3	0.4	1 M KOH	X37-50	Nickel foam	Carbon paper	1.0	80 ℃	10	5.0	[26]
2	0.4	1 M KOH	FAA-3-50	SUS	Carbon paper	1.0	50 ℃	50-200	5.0	[27]
