# Supplementary material for: Oxide-hybridized carbon as a catalyst support for efficient anion exchange membrane water electrolysis
Source: Nat Commun. 2025 Dec 12;16:11090. doi: 10.1038/s41467-025-65980-w (PMC12700888; doi:10.1038/s41467-025-65980-w)
Supplement: Supplementary file 2 — Description of Additional Supplementary Files [file 41467_2025_65980_MOESM2_ESM.pdf]

Description of Additional Supplementary Files

File Name: Supplementary Data 1:

Description: Atomic coordinates of the optimized structures employed in theoretical calculations.
